# CD46 is a cellular receptor for species D human adenovirus

**DOI:** 10.1128/mbio.01587-25

**Published:** 2025-09-22

**Authors:** Katarina Danskog, Fredrik Petersen, Lars Frängsmyr, Gabriel Gonzalez, Miriam Becker, Annasara Lenman, Niklas Arnberg

**Affiliations:** 1 Department of Clinical Microbiology, Umeå University 8075, Umeå, Sweden; 2 Institute for Vaccine Research and Development, Hokkaido University 12810, Hokkaido, Japan; 3 Institute for Experimental Virology, TWINCORE, Centre for Experimental and Clinical Infection Research, a joint venture between the Medical School Hannover and the Helmholtz Centre for Infection Research, Hannover, Germany; 4 Department of Biochemistry & Research Center for Emerging Infections and Zoonoses (RIZ), University of Veterinary Medicine Hannover, Hannover, Germany; 5 Wallenberg Centre for Molecular Medicine (WCMM), Umeå University 8075, Umeå, Sweden; 6 Umeå Centre for Microbial Research (UCMR), Umeå University, Umeå, Sweden; 7 The Laboratory for Molecular Infection Medicine Sweden (MIMS), Science for Life Laboratory (SciLifeLab), Umeå University, Umeå, Sweden; National Institutes of Health, Bethesda, Maryland, USA

**Keywords:** adenovirus, receptor-ligand interaction, CD46, virus-host interactions, vector biology

## Abstract

Human adenovirus species D (HAdV-D) contains two-thirds of all known HAdV types (116 in total) and is important as a vector in clinical applications. However, the broad tropism exhibited by several HAdV-D types poses challenges for their use as targeted gene delivery vectors. Since adenoviral tropism is largely governed by receptor usage, we aimed to determine the relative importance of known adenovirus receptors in mediating infection by different HAdV-D types. Here, we generated A549 single-cell CRISPR/Cas9 knockout clones of desmoglein 2 (DSG2), CD46, the coxsackievirus and adenovirus receptor (CAR), and cytidine monophosphate N-acetylneuraminic acid synthetase (CMAS; needed for biosynthesis of sialic acid-containing glycans), and assessed their relative importance for infection by 18 different HAdV-D types. We show that CD46 is the most important receptor for a majority of species D HAdVs. Minor changes in infection levels were noted with A549-ΔCAR and A549-ΔDSG2 cells, whereas A549-ΔCMAS cells displayed an increased susceptibility to infection. We proceed to show that HAdV-D types require CD46 for efficient attachment to A549 cells, and surface plasmon resonance analysis demonstrates that their hexon proteins bind CD46 in an avidity-dependent manner. Strategies to retarget HAdV-D vectors should thus consider hexon-CD46 interactions as a critical determinant of tropism, as CD46 is broadly expressed *in vivo*. These results increase our understanding of adenovirus-host interactions and will guide the development and targeting of vectors based on HAdV-D types.

**IMPORTANCE:**

Several human adenovirus species D (HAdV-D) types are currently used, or under development, as viral vectors for vaccines and gene delivery. However, the unusually broad tropism observed in many HAdV-D types limits their specificity and effectiveness as targeted vectors. Since tropism is largely governed by receptor usage, and previous studies have reported conflicting findings on receptor preferences within this species, clarifying receptor usage is essential. In this study, we systematically investigated receptor usage in 18 different HAdV-D types and identified CD46 as the primary receptor. Since CD46 is widely expressed across human tissues, our findings explain the broad cellular tropism of these viruses and provide valuable insight for the rational design and refinement of HAdV-D-based vectors.

## INTRODUCTION

Human adenoviruses (HAdVs) are non-enveloped DNA viruses with an icosahedral capsid composed of three major coat proteins: hexon, penton base, and fiber. The trimeric hexon proteins cover most of the capsid surface, and at each of the 12 vertices, a penton base is located, each bearing a protruding trimeric fiber terminating with a knob domain. HAdVs are classified into seven species (A–G), and denoted HAdV-C1, HAdV-D26, etc. To date, more than 116 human adenovirus types have been proposed, with 79 types belonging to species D (1). HAdVs exhibit distinct, but sometimes overlapping, tissue tropism and can cause diseases, such as conjunctivitis, keratitis, gastroenteritis, laryngitis, bronchiolitis, and pneumonia. Additionally, hepatitis and urethritis have been observed in immunocompromised individuals infected by HAdVs (2, 3). Many HAdV-D types have been isolated from the stool of AIDS patients or individuals undergoing stem cell transplants, where immunosuppression facilitates coinfections, leading to recombination and generation of new types (4–7). Some HAdV-D types, mainly HAdV-D8, -D64, -D37, -D53, -D54, and -D56, cause epidemic keratoconjunctivitis (EKC) (8), where HAdV-D8 and -D37 have also been linked to genital infections (9). HAdV-D15, -D64, -D29, -D30, -D37, and -D39 have been associated with respiratory infections, and HAdV-D56 was first isolated from a fatal case of neonatal pneumonia (10, 11). The broad tissue tropism within species D (12–15) likely reflects the engagement of multiple receptors. In combination with their ability to induce strong cellular immune responses and their overall low seroprevalence in humans, this has made them attractive candidates for vector development (16–19). Several HAdV types have been explored as viral vectors for clinical applications (20–26). For example, several HAdV-based viral vectors are currently under investigation to deliver Ebola virus vaccines in clinical trials (27–29).

HAdV infection typically begins with binding of the fiber knob to a primary receptor, followed by a lower-affinity interaction between the penton base and integrins on the cell surface to trigger internalization of the virus (30–32). This has been described for multiple HAdVs that use the fiber knob to engage desmoglein 2 (DSG2), CD46, the coxsackie and adenovirus receptor (CAR), and sialic acid-containing glycans (33–37). HAdV tropism can be further influenced by soluble molecules that bind both to HAdV particles and to cells, thereby facilitating interactions with additional receptors (38–40). Specific members of HAdV-D engage a wide array of cellular receptors (19, 36, 41–53), where receptor usage has primarily been studied in individual HAdV-D types. Some engage CD46, either via the fiber knob or the hexon protein, or by unknown mechanisms (41, 45, 54–57). Other reported receptors include sialic acid-containing glycans (HAdV-D26 and -D37) (36, 47–49, 58), including glycans that resemble those of the GD1a ganglioside (HAdV-D37) (50), CAR (HAdV-D26) (19), and heparan sulfate proteoglycans, which may act as decoy receptors for HAdV-D37 (51). HAdV-D26 alone has been linked to multiple receptors, including CD46 (41, 45), sialic acid (47, 59), scavenger receptor SR-A6 (52), αvβ3 integrin (53), and CAR (19). HAdV-D48 has been reported to engage both CAR and CD46 (18, 60); however, *in silico* and surface plasmon resonance (SPR) data indicate that the affinities of fiber knob interactions with these molecules are likely insufficient to support productive entry (61). The receptor usage of HAdV-D49 has not been clearly defined, but has been associated with promiscuity in tropism (16). Similarly, vectorized HAdV-D17 shows broad tropism but appears to depend on CD46 for cell entry in endothelial cells (62). Transduction by vectorized HAdV-D43 is also enhanced in Chinese hamster ovary (CHO) cells expressing CD46 (57). HAdV-D64 was recently shown to use CD46 as a receptor on conjunctival cells (56), while HAdV-D56 transduction improves in CHO cells expressing integrin α7, a marker for muscle cells (25). We recently identified a non-canonical entry pathway for HAdV-D56, involving direct binding of hexon to CD46 (45). To date, there are few comprehensive and systematic analyses of receptor preferences across species HAdV-D (63, 64), something that is particularly important given their increasing use as viral vectors.

To address this, we systematically knocked out the known, key adenovirus receptors in a human epithelial lung cell line (A549) and compared their relevance across 18 HAdV-D types. Most viruses showed markedly reduced infection in A549-ΔCD46 cells, indicating a strong preference for CD46. We demonstrate that HAdV-D binds CD46 with high affinity, predominantly via the hexon protein rather than the fiber knob. Although multiple receptors have been proposed for species D, our findings demonstrate that CD46 serves as the most important receptor in respiratory epithelial cells. We conclude that this is likely due to hexon-mediated avidity.

## RESULTS

### Generation and characterization of A549-KO cells to study receptor preferences for HAdV-D types

**Fig 1:**
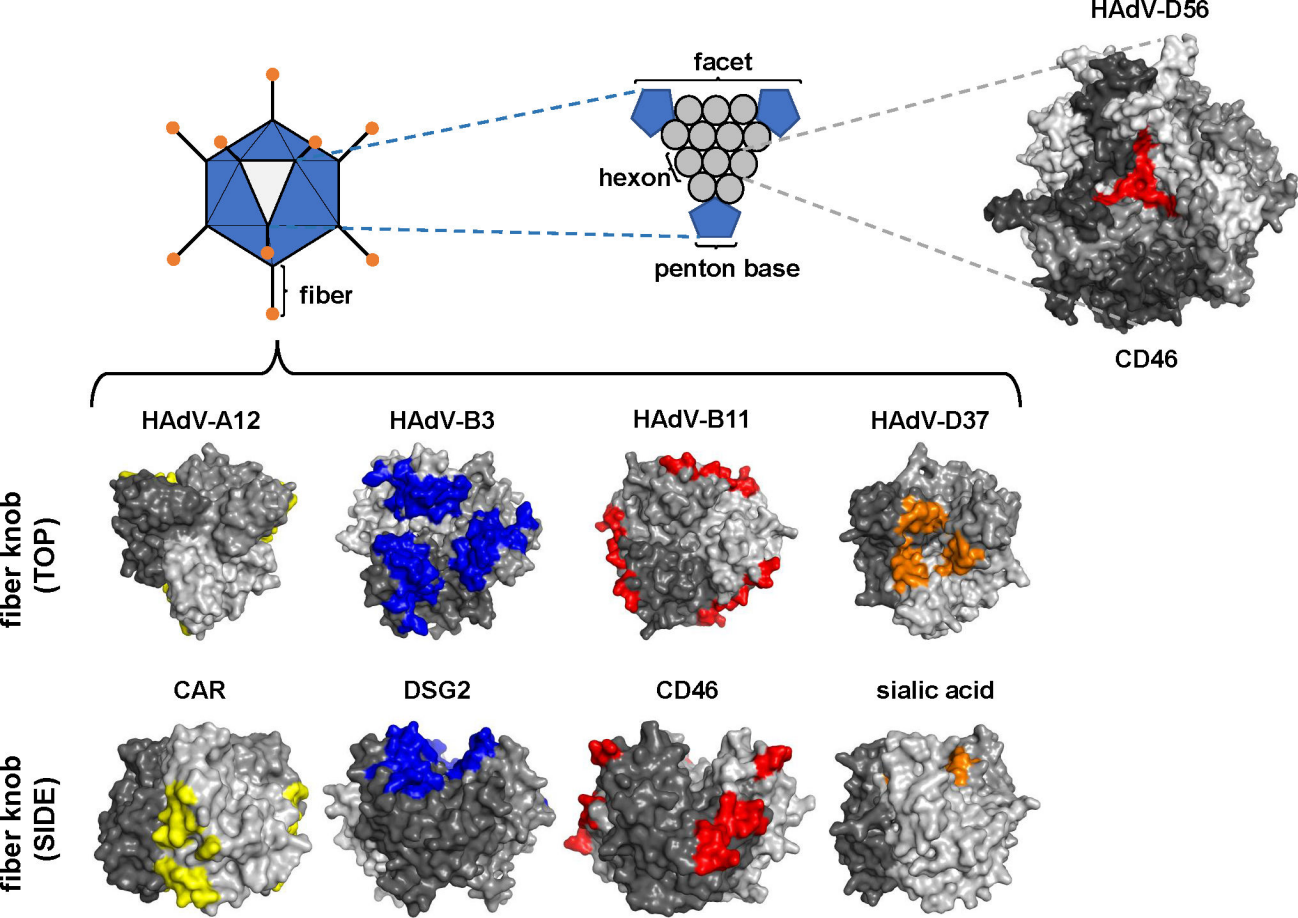
Structural comparison of predicted location of receptor-interacting surfaces of viral capsid proteins. Diagram of the proteins in the viral capsid, highlighting the trimeric fibers in the 12 vertices of the virion and the 240 trimeric hexon proteins distributed in the 20 facets forming the capsid. The top view of the predicted structure for the hexon protein of HAdV-D56 is shown, with the area shown to interact with CD46 colored in red (45). The top and side views of the fiber knobs for HAdV-A12, -B3, -B11, and -D37 are shown. The predicted binding sites for CAR (yellow), DSG2 (blue), CD46 (red), and sialic acid/cytidine monophosphate N-acetylneuraminic acid synthetase (CMAS) (orange) are shown and based on references 34, 48, 65, 66. Chains in the predicted trimers are colored on different gray *tones.*

The fiber knob is considered the main mediator of primary receptor attachment for AdVs, and the virus engages different areas of the knob depending on the receptor. DSG2 interacts with the center top of the HAdV-B3 trimeric knob, and sialic acid binds to the central cavity of the HAdV-D37 trimeric knob, whereas CAR and CD46 interact with the interface of the knob monomers (Fig. 1) (33–37). As for the interaction between HAdV-D56 hexon and CD46, the exact location of the attachment to the hexon is unknown, but increased density has been observed in the central cavity between the hexon trimers, indicating this as the binding site (Fig. 1).

To examine the impact of individual receptors on species D HAdV infection, we generated a panel of A549 CRISPR/Cas9 knockout cell lines, hereafter referred to as A549-ΔDSG2, A549-ΔCAR, A549-ΔCD46, and A549-ΔCMAS. A549 cells were chosen as our model system since they are widely used for wild-type HAdV propagation and naturally express the major HAdV receptors CAR, CD46, DSG2, sialic acid-containing glycans, as well as relevant integrins that are used as co-receptors by many HAdVs.

**Fig 2:**
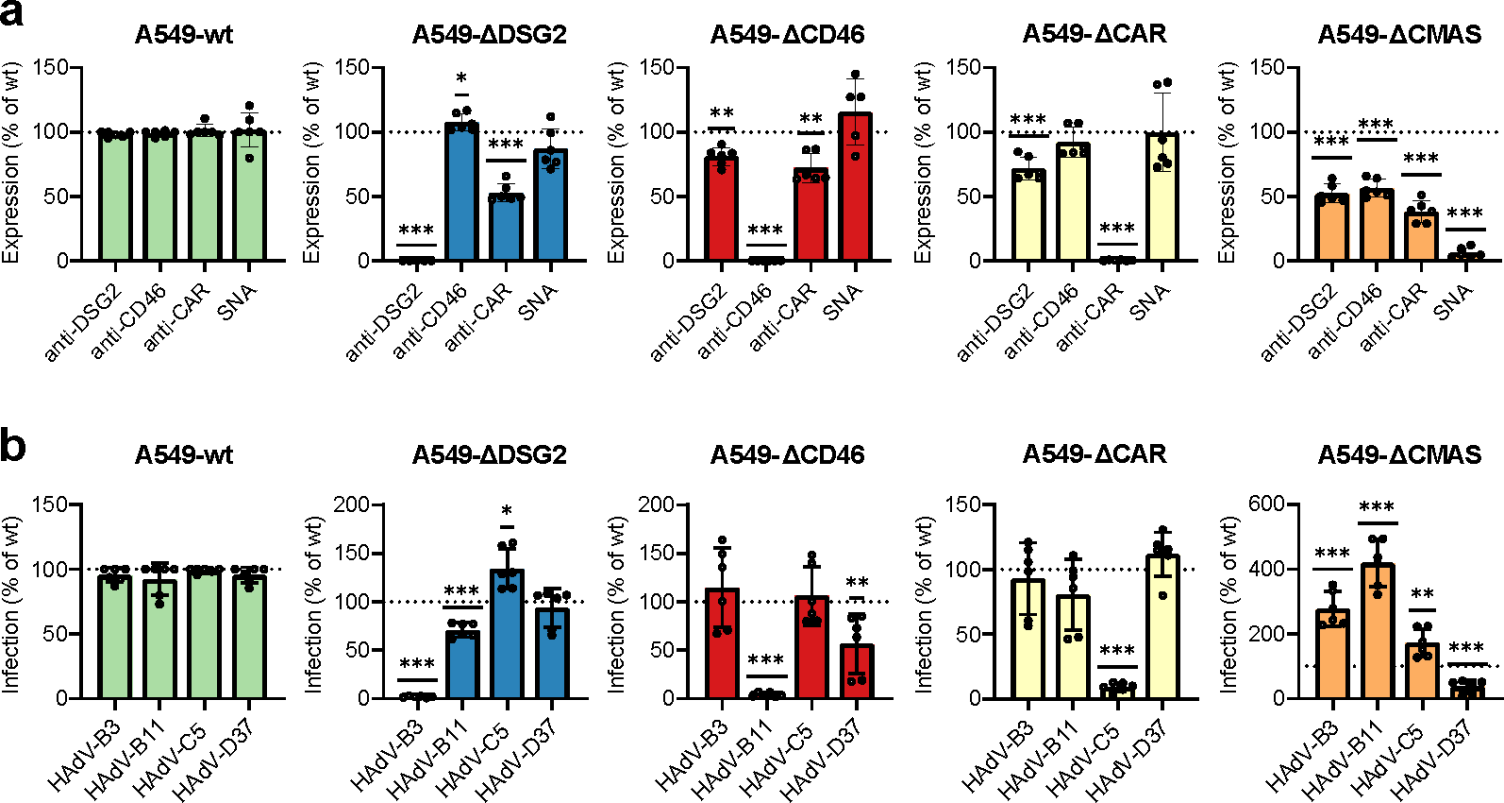
Characterization of CRISPR/Cas9 A549 knockout cell lines. (**a**) Cell surface expression of DSG2, CD46, CAR, and α2,6-linked sialic acid in A549-wt, -ΔDSG2, -ΔCD46, -ΔCAR, and -ΔCMAS, analyzed by flow cytometry. Expression is shown as a percentage relative to A549-wt levels. (**b**) Functional validation of receptor knockout by infection with control adenoviruses: HAdV-B3 (DSG2), HAdV-B11 (CD46), HAdV-C5 (CAR), and HAdV-D37 (sialic acid). Data in a and b represent the mean ± SD from three independent experiments. Statistical significance was determined using one-way analysis of variance (ANOVA) with Dunnett’s post-test and is compared against A549-wt; *, *P* < 0.03; **, *P* < 0.002; ***, *P* < 0.001.

Receptor knockouts were verified by flow cytometry and demonstrated complete loss of CAR, CD46, DSG2, and sialic acid expression in the respective knockout cell lines, while levels of the other receptors were largely maintained (Fig. 2a; Fig. S1a). Functional validation of receptor knockout was performed using HAdV-B3, -B11, -C5, and -D37, each known to use DSG2, CD46, CAR, and sialic acid, respectively, as primary receptors and bind to these receptors via the fiber knob domain (36, 67–70). In each case, infection was greatly reduced in the corresponding receptor-deficient cell line (Fig. S1b), and almost entirely abolished when normalized against wild-type A549 (A549-wt) infection (Fig. 2b), confirming effective disruption of receptor-mediated entry. We observed a minor reduction of CAR expression in A549-ΔDSG2 cells, which was not accompanied by reduced infection by HAdV-C5 in these cells. We also noted a slightly reduced infection by HAdV-B11 in these cells, despite a minor increase in CD46 expression. Similarly, we observed a modest reduction of both DSG2 and CAR on A549-ΔCD46, which was accompanied by a slight reduction of HAdV-D37 infection (but not of HAdV-B3 or HAdV-C5) in these cells. Analysis also displayed a small reduction of DSG2 expression on A549-ΔCAR cells, which was not accompanied by reduced HAdV-B3 infection. Finally, we validated loss of cell-surface sialylation in A549-ΔCMAS cells using *Sambucus nigra* lectin (SNA), which binds α2,6-linked sialic acid, and dropped to near-background levels. We noted that staining of DSG2, CD46, and CAR dropped to about 50%, yet there was an increased infection of HAdV-B3, -B11, and -C5, ranging between ca 50% and up to 400%. We assume that such relatively small deviations can be expected due to altered integrity of the plasma membrane, especially when molecules no longer contain any monosaccharides, which may affect interactions.

### HAdV-D types need CD46 for infection of A549 cells

**Fig 3:**
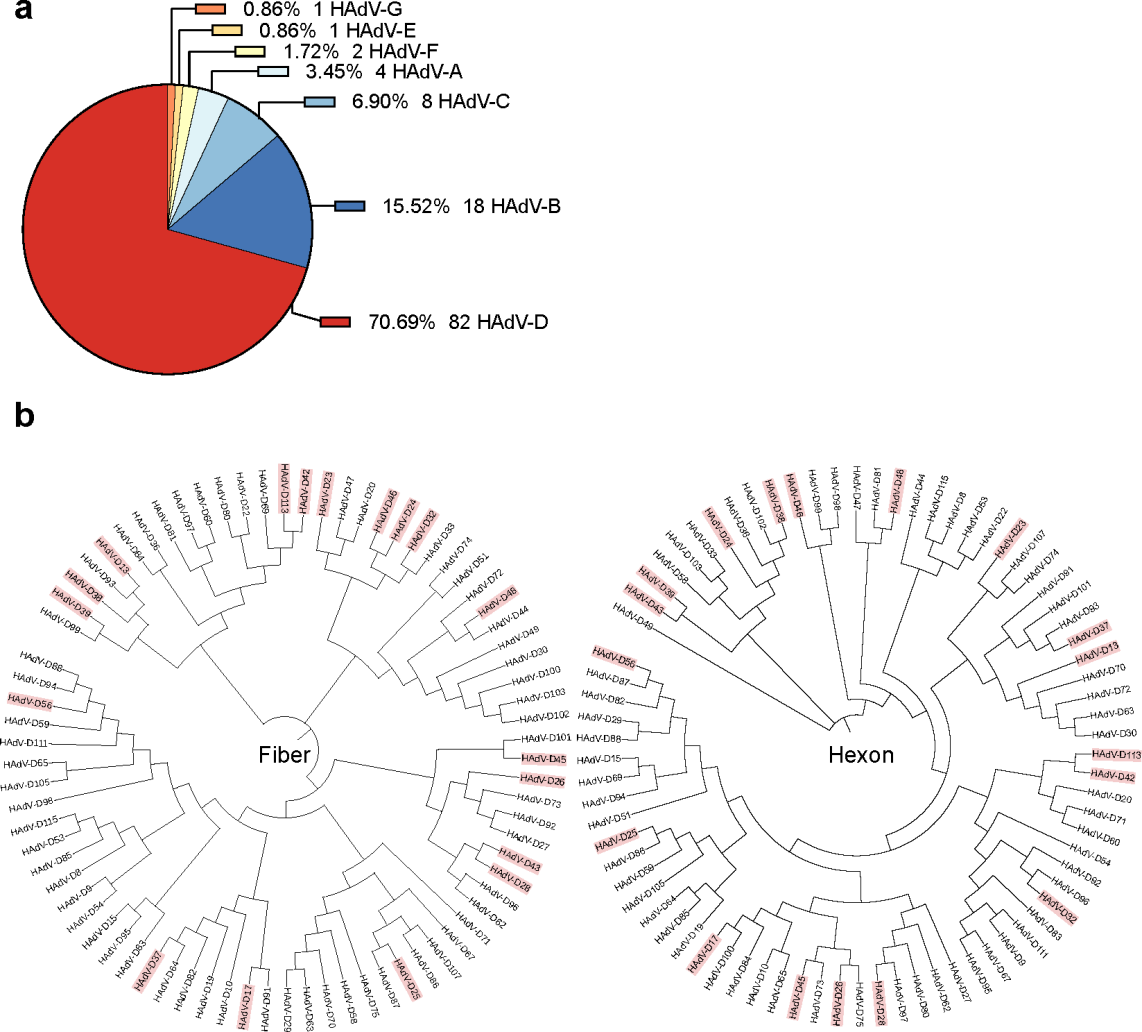
Species D is the largest HAdV species and displays high phylogenetic diversity. (**a**) Pie chart showing the number and percentage of HAdV types in each of the seven species (A–G). (**b**) Phylogenetic tree based on fiber (left) and hexon (right) amino acid sequences of all known species D HAdV, generated using Clustal Omega (71) and iTOL version 7.1 (72). HAdV-D types used in this study are highlighted in red.

To investigate the role of CD46 in the context of the very large and diverse species HAdV-D group, of which many have been explored as vectors for clinical applications, we next infected wild-type and A549-KO cell lines with 18 different HAdV-D types: HAdV-D13, -D17, -D23, -D24, -D25, -D26, -D28, -D32, -D37, -D38, -D39, -D42, -D43, -D45, -D46, -D48, -D56, and -D113. Over 70% of all HAdV types belong to species D (Fig. 3a). Species D HAdVs display a great genomic diversity in the fiber knob region (61). We selected types from different branches of a phylogenetic tree based on the fiber and hexon amino acid sequences of HAdV-D types (Fig. 3b), ensuring that the broad genetic diversity within species D was well represented. To establish a baseline for comparison, viruses were titrated on A549-wt to achieve an infection level of approximately 10%–20%.

**Fig 4:**
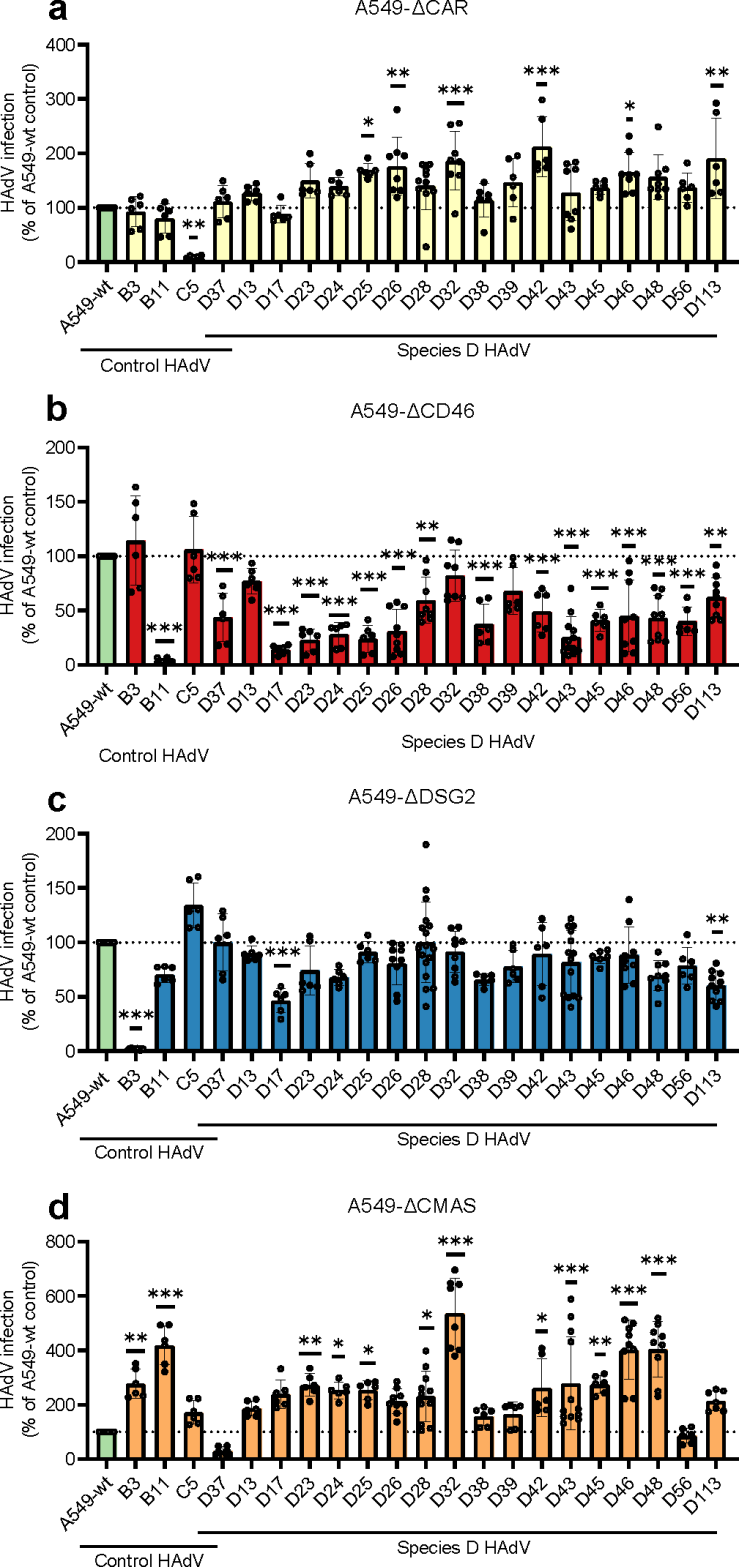
Screening of HAdV-D types on CRISPR/Cas9 A549 knockout cell lines. A549 cells were infected with HAdV-B3, -B11, -C5, and 18 HAdV-D types and subsequently analyzed for production of viral hexon protein by immunostaining 44 h post-infection. Each virus was titrated on A549-wt cells to give 10%–20% infected cells and subsequently used to infect (**a**) A549-ΔDSG2, (**b**) A549-ΔCD46, (**c**) A549-ΔCAR, and (**d**) A549-ΔCMAS cells. Data is shown as percent infection normalized to A549-wt, based on at least three independent experiments. Results are presented as mean ± SD. Statistical significance was determined using one-way ANOVA with Dunnett’s post-test and is compared against A549-wt; *, *P* < 0.05; **, *P* < 0.005; ***, *P* < 0.001.

We observed some reduction of infection by some types in A549-ΔDSG2 cells (Fig. 4a). Even if these reductions were modest in most cases, they were statistically significant for several D types: HAdV-D13, -D17, -D23, -D24, -D26, -D38, -D39, -D43, -D45, -D48, -D56, and -D113. The most pronounced effect was observed for HAdV-D17, where infection was reduced by more than 50%. Nearly all HAdV-D types infected A549-ΔCD46 cells less efficiently as compared to A549-wt cells (Fig. 4b), with the largest decrease seen for HAdV-D17, -D23, -D24, -D25, -D26, -D38, and -D43 for which infection was reduced by >50%. HAdV-D32 was the only D type that infected A549-ΔCD46 as efficiently as wild-type cells. No HAdV-D types infected A549-ΔCAR cells less efficiently, suggesting that CAR is not essential for infection by HAdV-D types (Fig. 4c). We observed slightly higher infection levels in CAR-knockout cells than in wild-type cells. Notably, almost all tested HAdV-D types infected sialic-acid-deficient A549-ΔCMAS better relative to wild type (Fig. 4d), except for HAdV-D37 and -D56, which are both EKC-causing viruses (73). In contrast to the results obtained with A549-ΔCD46 cells, none of the other knockouts (A549-ΔCAR, -ΔDSG2, or -ΔCMAS) showed a comparable reduction in infectivity across species D viruses, suggesting that CD46 is a preferred receptor for most species D HAdVs. This observation aligns with earlier reports identifying several different receptors for individual HAdV-D types (19, 36, 41–45, 47–53, 58).

### Attachment of HAdV-D types to A549-ΔCD46 cells largely corresponds to infection

**Fig 5:**
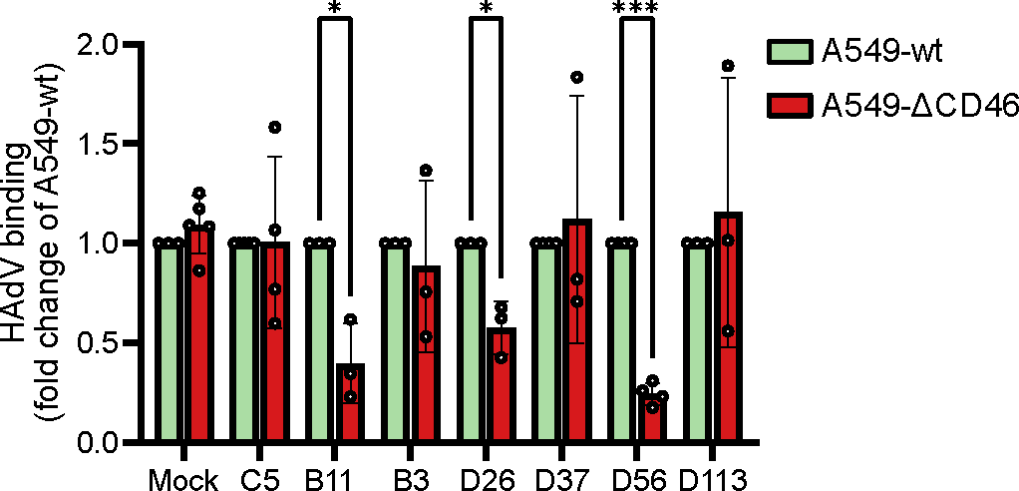
HAdV attachment is reduced to A549-ΔCD46 cells compared to A549-wt. HAdVs were allowed to bind to A549 cells for 60 min on ice, and the amount of viral DNA bound to cells was quantified by qPCR. Binding to A549-ΔCD46 cells was normalized against binding levels to A549-wt cells. Data are from at least three independent experiments, presented as mean ± SD. Statistical significance was determined with an unpaired *t*-test; *, *P* < 0.03; ***, *P* < 0.001.

As the infectivity of the HAdV-D types was markedly reduced in A549 cells lacking CD46, we next wanted to determine whether the absence of CD46 also affected viral attachment. We performed quantitative PCR (qPCR)-based attachment experiments using four HAdV-D types (HAdV-D26, -D37, -D56, and -D113), along with control viruses HAdV-B3, -B11, and -C5. As expected, attachment of HAdV-B11 was significantly reduced on A549-ΔCD46, while no reduction was observed for HAdV-B3 or C5 (Fig. 5). Consistent with the infectivity data, HAdV-D26 and -D56 showed reduced binding to CD46-knockout cells compared to wild-type A549, whereas attachment of HAdV-D37 was unaffected by the absence of CD46 (Fig. 5). Interestingly, HAdV-D113 binding was not significantly decreased in A549-ΔCD46 cells, despite its reduced infectivity (Fig. 4b and 5).

### HAdV-D binds to CD46 via hexon and by avidity-dependent mechanisms

**Fig 6:**
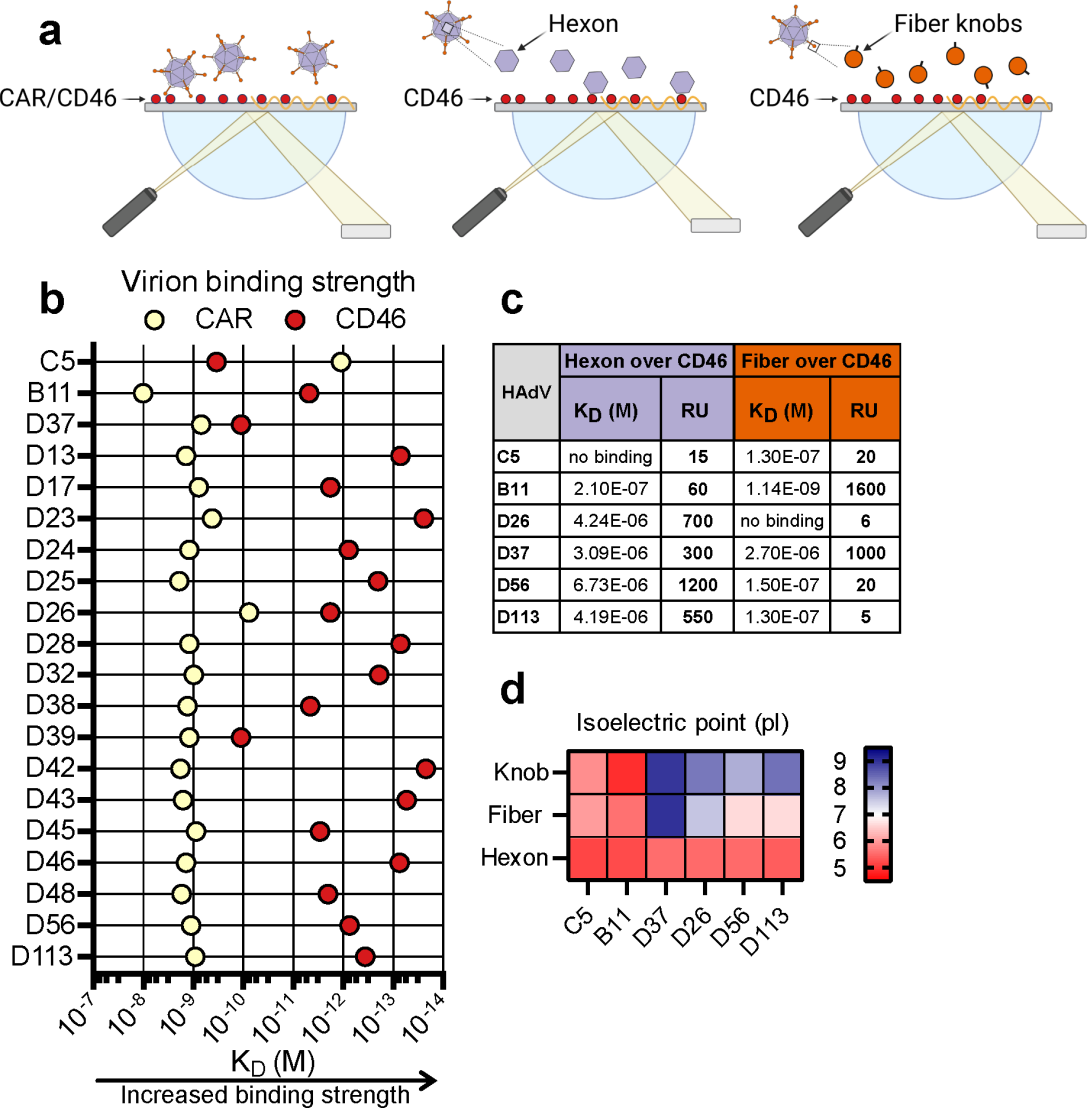
HAdV-D binds directly to CD46 via the hexon. (**a**) Experimental set-up of SPR experiments with whole virus (left), hexon (middle), or fiber knob (right) as analyte over immobilized CD46 or CAR. (**b**) Binding affinities of purified HAdV-D type virions to immobilized CAR or CD46, measured by SPR and shown as binding constants (K_D_). (**c**) Table of SPR binding constants (K_D_) and response units (RU) between purified HAdV-D hexon or fiber knobs over immobilized CD46. (**d**) Isoelectric points (pI) of hexon and fiber knob proteins from the HAdVs used in (**c**), calculated using Expasy ProtParam (76). Experiments in (**b**) and (**c**) were performed once.

Given the reduced infection of HAdV-D types in A549-ΔCD46 cells, we next wanted to characterize their binding affinity and mode of interaction with CD46. We performed SPR analysis using immobilized CD46 or CAR and applied either whole virions or purified capsid proteins as analyte (Fig. 6a). Binding profiles were compared to the control viruses HAdV-C5 and -B11. As expected, HAdV-C5 virions bound strongly to CAR (1.1 pM), and similarly, HAdV-B11 bound almost as strongly to CD46 (4.8 pM) (Fig. 6b). Most HAdV-D types bound to CAR with similar affinities in the nanomolar range, although HAdV-D26 displayed a slightly stronger affinity at 76 pM. In contrast, all HAdV-D types bound CD46 with markedly higher affinity, ranging from 100 pM (HAdV-D37 and D39) to low femtomolar levels (HAdV-D23 and D42) (Fig. 6b). We have previously reported that HAdV-D56 binds CD46 via the hexon protein (45), suggesting this might also be the case for other HAdV-D types. To investigate this, we performed additional SPR experiments using purified fiber knobs or hexon proteins from selected viruses (HAdV-C5, -B11, -D26, -D37, -D56, and -D113) as analytes over immobilized CD46. In agreement with previous studies, neither hexon nor fiber knob from HAdV-C5 interacted with CD46, whereas HAdV-B11 bound CD46 exclusively via the fiber knob (Fig. 6c) (70, 74). In contrast, all tested HAdV-D viruses bound CD46 via the hexon protein, with HAdV-D37 being the only one that also showed binding via the fiber knob, as indicated by the response units (Fig. 6c). Although affinity data indicated that the binding strength to CD46 was strong for both hexon and fiber, the response units revealed the hexon to be a more potent binder of CD46. Since CD46 is rich in sialic acid (75), and HAdV-D37 is known to bind sialic acid through charge-dependent interactions, we propose that the observed interaction between the HAdV-D37 fiber knob and CD46 occurs via charge-dependent interactions with sialic acid. To further investigate electrostatic interactions, we calculated the isoelectric point (pI) of the hexon and the fiber knob of HAdV-C5, -B11, -D37, -D26, -D56, and -D113. HAdV-D37 displayed the highest pI (8.97) in the knob domain (Fig. 6d). Although none of the other HAdVs had an equally high pI, all HAdV-D type knobs were positively charged at neutral pH, with pI values ranging from 7.8 to 8.4 (Fig. 6d).

## DISCUSSION

Species D is the largest group within the HAdV family and has attracted considerable interest as a viral vector, in part due to its low seroprevalence and broad tissue tropism (16–19, 77). This wide tropism mirrors the fact that several cellular receptors have been proposed for HAdV-D (36, 41, 43–45, 47–53, 58), yet their preferential receptor has remained unclear. In this study, we demonstrate that HAdV-D types preferentially use CD46 for entry and infection, rather than CAR, DSG2, or sialic acid-containing glycans. Notably, 17 out of the 18 HAdV-D types tested required CD46 for efficient infection of A549 cells, with HAdV-D32 being the only exception, and with HAdV-D39 depending weakly on CD46 (Fig. 4). CD46 dependence for infection correlated in most cases well with avidities (Fig. 6), including low avidities between HAdV-D37 and D39 and CD46, but also with some exceptions, such as relatively high avidity between HAdV-D32 and CD46. We assume that for some HAdV-D types, additional interactions with other host cell molecules can affect the dependence of CD46, such as HAdV-D37 and sialic acid. We further show that the interaction between HAdV-D types and CD46 is primarily mediated by the hexon protein rather than the fiber knob, and our SPR analyses indicate that this occurs through an avidity-dependent binding mechanism. As CD46 is expressed on all nucleated human cells, this could explain the broad tropism observed for HAdV-D types *in vivo*. However, infection was not completely abolished in A594-ΔCD46 cells, suggesting that additional cell surface molecules may either facilitate infection or compensate for the absence of CD46. It’s important to note that our study was conducted using a lung epithelial cell line, which provides a controlled model for comparing receptor usage. Receptor expression and usage can vary across different tissues and cell types, and thus, some HAdV-D types might behave differently in other cellular contexts or *in vivo*. In a study by Turner et al. (78), HAdV-D26 infection of A549 cells resulted in rounding and detachment of infected cells (although still viable), a phenotype we did not observe in our experiments. Yet, we cannot exclude the presence of some detached cells, which may impact the results. Future studies could extend this knockout approach to cell lines representing ocular or gastrointestinal epithelia or use advanced primary models to confirm the dominance of CD46 across different contexts. Together, our findings position CD46 as a central entry receptor for species D HAdVs, while also highlighting the potential for receptor redundancy *in vivo*.

CD46 dependence was robust across most HAdV-D types. HAdV-D32 has not been extensively studied, but has been associated with a case of encephalitis (79), a rare tropism for HAdVs. Its CD46 independence highlights the possibility of alternative receptor usage and warrants further investigation to clarify its entry mechanism. These results aligned well with those described by Tsoukas et al. (64), using a similar approach with CD46-KO A549 cells and a library of species D HAdVs, and with those described by Chen et al. (80), also using a panel of species D HAdVs but with B-cell cancers as targets.

In virus attachment experiments, HAdV-D26 and -D56 presented reduced attachment to A549-ΔCD46 cells, whereas HAdV-D37 and HAdV-D113 did not. For HAdV-D37, this could be explained by fiber knob interactions with sialic acid (36, 58), masking other receptor effects. Although HAdV-D113 binding to A549-ΔCD46 was unaffected, we observed a reduced infectivity, suggesting that other molecules may mediate initial attachment, or that the hexon-CD46 interaction contributes more substantially to entry via avidity-dependent mechanisms that only become evident during infection. As an example, HAdV-B3 and -B7, which bind with high affinity to DSG2, can also use CD46 if it is present in high enough density through increased avidity (81). All HAdV-D types tested displayed an exceptionally high binding strength to CD46, primarily in the femtomolar range, compared to nanomolar-range binding to CAR, demonstrating the ability of species D HAdVs to directly engage with CD46. Further analysis of capsid proteins revealed that -D26, -D37, -D56, and -D113 interact with CD46 via the hexon protein, with HAdV-D37 also being able to bind via the fiber knob, possibly due to interaction with sialic acid present on CD46. Interestingly, although the overall virion pI for these types was relatively high (7.8–8.4), indicating a net positive charge at neutral pH, their hexon proteins all had a pI near 5.5, suggesting that charge is not a primary determinant of the hexon-CD46 interaction.

Nearly all HAdV-D types infected sialic acid-lacking A549-ΔCMAS more efficiently than wild-type cells, with the exception of EKC-causing HAdV-D37 (reduced) and HAdV-D56 (unchanged) (73). We could not refer this to increased expression of, e.g., CD46 in A549-ΔCMAS cells, as these cells rather displayed a slight decrease in expression of CD46, CAR, and DSG2. We speculate that this may be due to altered binding of antibodies to each receptor after sialic acid removal, since all three receptors are glycosylated (69, 75, 82). Infection with HAdV-D types in A549-ΔCMAS cells ascertained that loss of sialylation had a profound effect on infectivity. These results confirm previous data demonstrating that HAdV-D37 uses sialic acids as a primary receptor (36, 58) and that HAdV-D56 infection in human corneal epithelial cells is unaffected by enzymatic removal of sialic acid (25, 49). Vectors equipped with HAdV-D26 fiber knobs engage sialylated glycans via the fiber knob and use this as a primary receptor (47). The knob of HAdV-D26 has local pockets with a high isoelectric point, facilitating a charge-dependent interaction with sialic acid. Interestingly, in a recent comprehensive analysis by Mundy et al. (59), it was reported that many species D HAdVs have fiber knobs capable of binding sialic acid, and that enzymatic removal of sialic acid reduced infection by pseudotyped HAdV-C5 vectors carrying D-type knobs. In contrast, we observed increased infectivity of native HAdV-D virions in the absence of sialic acid. This discrepancy may be explained by the multivalent contribution of HAdV-D hexons in our system: In wild-type virions, HAdV-D type hexon-CD46 interactions likely compensate for, and even outweigh, fiber-sialic acid interactions. In pseudotyped systems using species C HAdV backbones, whose hexons bind weakly or not at all to CD46, fiber knob:sialic acid interactions become more critical, and neuraminidase treatment reduces infection of such constructs. We also speculate that depending on the context, HAdV-D fiber:sialic acid interactions may either be functional/productive as in the context of HAdV-C-based vectors or be of a decoy-receptor type interaction, where removal of the decoy receptor facilitates infection. Thus, these studies together suggest a model in which species D HAdVs can use both sialylated glycans (via the fiber knob) and CD46 (via the hexon), with the latter pathway likely playing a dominant role in infection by native viruses.

None of the tested species D members showed reduced infectivity in A549-ΔCAR cells; instead, most exhibited slightly increased infection. We could not relate this to altered expression of other receptors in these cells, and the expression of these receptors was largely intact. Nevertheless, as CAR primarily functions as a tight junction adhesion molecule, its absence may affect cell surface architecture in a way that favors access to other, functional receptors, such as CD46 or decrease access to other molecules that may act as decoy receptors (51). Others have seen decreased transduction of AdV vectors from species D in both A549-ΔCD46 and A549-ΔCAR cells (64), implying the necessity for both surface proteins to be present during transduction. Some HAdV-D types, such as HAdV-D9, -D26, and -D48, do bind CAR, but with significantly lower affinity than members of other species like HAdV-C5, -A12, and -F41 (42, 61, 83). Although some HAdV-D types have been suggested to use CAR as a receptor (19, 43), and HAdV-D37 can bind CAR with nanomolar affinity (44), this does not appear to translate into infectious entry, possibly due to the short, rigid nature of its fiber shaft and the presence of a semi-flexible loop in the knob domain that introduces steric hindrance (61, 84). Collectively, our results suggest that CAR is not a major receptor for species D HAdVs. This opens up alternative roles of the fiber-CAR interaction. Walters et al. (85) proposed that excess of HAdV-C2 fibers produced during infection are secreted at the basolateral side and disrupt intercellular CAR dimers, which facilitates transmission of progeny virions to neighboring cells, and from the site of infections to new target sides. It is tempting to speculate that species D HAdVs that use hexon-CD46 interactions as a main mechanism for cell entry have preserved a similar function of the fiber protein.

DSG2 has previously only been described as a receptor for some HAdV-B viruses (67), and its role for species D HAdVs is unclear. In our study, we did not observe any major effect on infectivity of HAdV-D types in A549-ΔDSG2 cells. The largest effect was seen for HAdV-D17, for which infectivity was reduced by around 50%, suggesting a potential, though minor, role. Given that species B viruses interact with DSG2 via the fiber knob, it is possible that some HAdV-D types retain this ability through structural similarity.

The current study is limited to the usage of A549 cells, which do not fully represent the tropism of all species D HAdVs. However, other model systems using human endothelial, conjunctival peripheral blood mononuclear cells, and transgenic mice expressing human CD46 all support a role of CD46 in species D HAdV infection (18, 23, 41, 56, 62).

Historically, CAR has been considered the primary receptor for HAdVs, except for species B, which uses CD46 or DSG2, and for a few EKC-causing species D types that use sialic acid. Here, we show that CD46 is an important receptor for a majority of species D HAdVs, an important insight, given that this species accounts for over two-thirds of all described human adenovirus types. The broad expression of CD46 may explain the wide tropism observed and could be advantageous for several vaccine administration routes conferring systemic immunity. However, it could also present challenges in cases where off-target transduction needs to be minimized. Moreover, strategies to retarget HAdV-D vectors via fiber modifications should also consider hexon-CD46 interactions as critical determinants of tropism and specificity. In summary, our findings redefine the receptor landscape for species D HAdVs and provide a deeper understanding of their cellular tropism. This knowledge will be useful for the design, targeting, and refinement of viral vectors based on HAdV-D types.

## MATERIALS AND METHODS

### Cells and viruses

A549 cells (ATCC) were maintained in DPH (Dulbecco’s modified eagle medium [Sigma] with 100 µg/mL Penicillin + 100 U/mL Streptomycin [Gibco] and 20 mM Hepes [Fisher]) supplemented with 10% fetal bovine serum (FBS) (HyClone). Wild-type human adenoviruses were propagated in A549 cells and purified on a cesium chloride (CsCl) gradient as described previously (86, 87). The fraction loaded on top of the CsCl gradient (hereon called “top phase”) was collected after centrifugation for purification of hexon proteins. Purified virions were stored in phosphate-buffered saline (PBS) with 10% glycerol at −80°C until further use.

### Purification of adenovirus hexon

The top phase from virus purification was collected, and buffer exchange was done using a 50 kDa MWCO Amicon spin column to 20 mM Hepes, pH 7.4. Using an NGC chromatography system (BioRad) with the ChromLab software, the sample was bound to an anion exchange column (HiTrap Q FF, Cytiva). The sample was eluted in a stepwise gradient of 10%–50% NaCl in 5% steps. The fraction containing hexon was identified by SDS-PAGE. This fraction was concentrated on a 50 kDa MWCO Amicon spin column to 500 µL total volume and applied on a Superose 6 increase size exclusion column (10/300 GL) and eluted in GF buffer (20 mM Hepes, pH 7.4, 150 mM NaCl). The purity of the hexons was further evaluated with SDS-PAGE, and samples were finally stored in GF buffer at −20°C until further use.

### Design of gRNAs and CRISPR/Cas9 plasmid construction

Using the Benchling CRISPR/Cas9 gDNA design tool (http://benchling.com), sequences were generated targeting the genes *DSG2*, *CD46* (*MCP*), *CXADR* (*CAR*), and *CMAS* (cytidine monophosphate N-acetylneuraminic acid synthetase). Three gRNA sequences for each gene were chosen, and all the oligos used are listed in Table 1. To generate CRISPR plasmids, gRNAs were cloned into the Bbs1 restriction site of pSpCas9 (BB)-2A-Puro (PX459) (#48138; Addgene) according to the protocol by Ran et al. (88). Plasmids were transformed into Stbl2 cells by heat shock for 30 s at 42°C, and single colonies were selected. All plasmids were confirmed for successful ligation by Sanger sequencing (Eurofins Genomics).

**TABLE 1:** gRNAs targeting DSG2, CD46, CXADR, and CMAS

gRNA sequences
DSG2_1 R 5’-aaacGGGACGCGCGTACGCCCTGC-3′ DSG2_1 F 5’-caccgCAGGGCGTACGCGCGTCCC-3′ DSG2_2 R 5’-aaacCTTCCAACGTTAAAGCAGATC-3′ DSG2_2 F 5’-caccgATCTGCTTTAACGTTGGAAG-3′ DSG2_3 R 5’-aaacCGGGGGCGGTGATCCAGGCGC-3′ DSG2_3 F 5’-caccgCGCCTGGATCACCGCCCCCG-3′
CD46_1 R 5’-aaacCCGCGAGTGTCCCTTTCCTTC-3′ CD46_1 F 5’-caccgAAGGAAAGGGACACTCGCGG-3′ CD46_2 R 5’-aaacTACTATGAGATTGGTGAACGC-3′ CD46_2 F 5’-caccgCGTTCACCAATCTCATAGTA-3′ CD46_3 R 5’-aaacTGCAAATGGGACTTACGAGTC-3′ CD46_3 F 5’-caccgACTCGTAAGTCCCATTTGCA-3′
CAR_1 R 5’-aaacCGCGGGTTGGAGACGGAGGTC-3′ CAR_1 F 5’-caccgACCTCCGTCTCCAACCCGCG-3′ CAR_2 R 5’-aaacCCGCCAGGTGCTTAATGTTC-3′ CAR_2 F 5’-caccgAACATTAAGCACCTGGCGG-3′ CAR_3 R 5’-aaacTCGAAACTGATGGCGTCTCAC-3′ CAR_3 F 5’-caccgTGAGACGCCATCAGTTTCGA-3′
CMAS_1 F 5’-caccgCGCGGGTTGGAGACGGAGGT-3′ CMAS_1 R 5’-aaacCACCTCCGTCTCCAACCCGCG-3′ CMAS_2 F 5’-caccgCCGCCAGGTGCTTAATGTTC-3′ CMAS_2 R 5’-aaacCGAACATTAAGCACCTGGCGG-3′ CMAS_3 F 5’-caccgTCGAAACTGATGGCGTCTCA-3′ CMAS_3 R 5’-aaacCTGAGACGCCATCAGTTTCGA-3′

### Generation of CRISPR/Cas9 knockout A549 cell lines

A549 cells were transfected with the three CRISPR plasmids targeting a single gene using Lipofectamine 3000 (ThermoFisher) according to the manufacturer’s instructions, and selection of transfected cells was done using puromycin (ThermoFisher) at 1 µg/mL for 72 h. Following selection, cells were seeded at low density in petri dishes from which single clones were isolated and subsequently propagated for further use.

### Verification of CRISPR/Cas9 knockout A549 cells by flow cytometry

Cells were detached using PBS with 0.05% ethylenediaminetetraacetic acid (EDTA) and reactivated in growth medium for 60 min at 37°C on a tipping board. Cells were washed with PF buffer (PBS with 2% FBS) and single stained for cell surface expression of adenovirus receptors DSG2, CAR, CD46, or sialic acid using, respectively, either mouse IgG anti-DSG2 (Santa Cruz Biotechnology AH12.2) 1:100, mouse IgG anti-CAR (Merck 05-644) 1:2,000, mouse IgG2ak anti-CD46(MCP)-FITC (Ancell 197-040) 1:50, or SNA conjugated with biotin (Vector laboratories B1305) 1:500, for 30 min on ice. After washing away unbound antibodies with PF buffer, cells were incubated with either an Alexa Fluor-488 conjugated anti-mouse IgG (H+L) secondary antibody (Invitrogen) diluted 1:1,000, or Alexa Fluor-488 conjugated streptavidin (Invitrogen) diluted 1:1,000 for 30 min on ice. Samples were analyzed for fluorescence intensity using a BD Accuri C6 instrument (Becton Dickinson).

### Adenovirus infection assay

A549 cells (wild type and KO) were seeded at a density of 25,000 cells/well in black, clear-bottomed 96-well plates (Greiner Bio-One). After 24 h, cells had formed confluent monolayers and were washed with DPH and subsequently infected with HAdVs diluted in DPH for 60 min at 37°C in a humidified incubator with 5% CO_2_. After washing away unbound virions, cells were left for 48 h in DPH with 2% FBS before being fixed with 4% PFA for 15 min at RT and permeabilized with 100% MeOH for 10 min at −20°C. Cells were stained for the presence of free hexon protein using a mouse IgG1 anti-adenovirus hexon antibody (MAB8052, Millipore) diluted 1:500 in PBS for 30 min at RT, and subsequently anti-mouse IgG (H+L) Alexa Fluor-488 conjugated secondary antibody (ThermoFisher) diluted 1:1,000 in PBS for 30 min at RT. With 15 min remaining, Hoechst 33342 (ThermoFisher) diluted 1:10,000 was added as nuclear stain. Afterward, cells were imaged with a Cytation 5 microplate reader (Agilent) and analyzed for the total number of cells and number of infected cells (adenovirus hexon positive) using the BioTek Gen5 software (Agilent).

### Adenovirus attachment assay

A549 cells (wild type and ΔCD46) were seeded at a density of 200,000 cells/well in 12-well plates (VWR, Avantor). After 24 h, cells had formed a confluent monolayer and were rinsed with cold DPH. On ice, 5,000 virus particles/cell of HAdV in DPH were added for 60 min. Cells were rinsed three times with cold PBS, and samples were harvested by cell lysis. Total DNA was isolated using a NucleoSpin Tissue DNA extraction kit (Macherey-Nagel, 740952). HAdV genomes were quantified by qPCR using qPCRBIO Probe Mix Lo-Rox (QPCRBIO, PB20.21) with PCR conditions being 1 cycle of 95°C for 2 min, 40 cycles of 95°C for 5 s and 65°C for 30 s. Relative gene expression was calculated by the 2-ΔΔCT method, where the HAdV gene expression was normalized against GAPDH expression in each cell type. Primers and probe detecting vial hexon were 5′-CWTACATGCACATCKCSGG-3′ forward primer; 5′-CRCGGGCRAAYTGCACCAG-3′ reverse primer; 5′-[6FAM]AGGACGCCTCGGAGTACCTGAGCCCCG[TAMRA]-3′ probe, and for detection of GAPDH, the TaqMan Gene Expression Assay kit was used (Thermo Fisher, 4331182, Hs02758991_g1).

### Surface plasmon resonance analysis of whole virus or virus proteins binding to CD46 and CAR

CM5 sensor chips, an amine-coupling kit, and HBS-EP+ buffer (10 mM HEPES, 150 mM NaCl, 3 mM EDTA, and 0.005% [vol/vol] surfactant P20 [pH 7.4]) were all purchased from GE Healthcare. All SPR experiments were performed at 25°C in HBS-EP+ running buffer. Data were collected with a Biacore T200 instrument at a rate of 1 Hz. CD46 and CAR were coupled to the CM5 sensor chip by amine coupling reactions according to the manufacturer’s instructions, aiming for an immobilization density of 2,390 to 2,640 for CD46 and 2,380 to 2,870 for CAR resonance units (RU). The surface of the upstream flow cell was used as a reference and was subjected to the same coupling reaction in the absence of protein. The analytes, virion, hexon, or fiber knob, were serially diluted in running buffer to prepare a twofold concentration series with HAdV (50–12.5 µg/mL), hexon (100–6.25 µg/mL), and fiber knob (50–12.5 µg/mL). Samples were injected as a series over reference and experimental biosensor surfaces for 120 s at a flow rate of 30 µL/min. Blank samples containing only running buffer were also injected under the same conditions to allow for double referencing. After each cycle, the biosensor surface was regenerated with a 60 s pulse of 10 mM Tris-glycine (pH 1.5) at a flow rate of 30 µL/min.

### Statistics

Experiments were performed at least three times in duplicates or triplicates. Results are expressed as means ± SD, and student’s *t*-test or one-way analysis of variance (ANOVA) was performed using GraphPad Prism, version 10.4.2 for Windows. *P* values of <0.05 were considered statistically significant.

## SUPPLEMENTAL MATERIAL

Fig. S1 mbio.01587-25-s0001.docx Complementary (raw data) to Fig. 2.

ASM does not own the copyrights to Supplemental Material that may be linked to, or accessed through, an article. The authors have granted ASM a non-exclusive, world-wide license to publish the Supplemental Material files. Please contact the corresponding author directly for reuse.
