# Supplementary material for: CD46 is a cellular receptor for species D human adenovirus
Source: mBio. 2025 Sep 22;16(11):e01587-25. doi: 10.1128/mbio.01587-25 (PMC12607895; doi:10.1128/mbio.01587-25)
Supplement: Fig. S1 — Complementary (raw data) to Fig. 2. [file mbio.01587-25-s0001.docx]

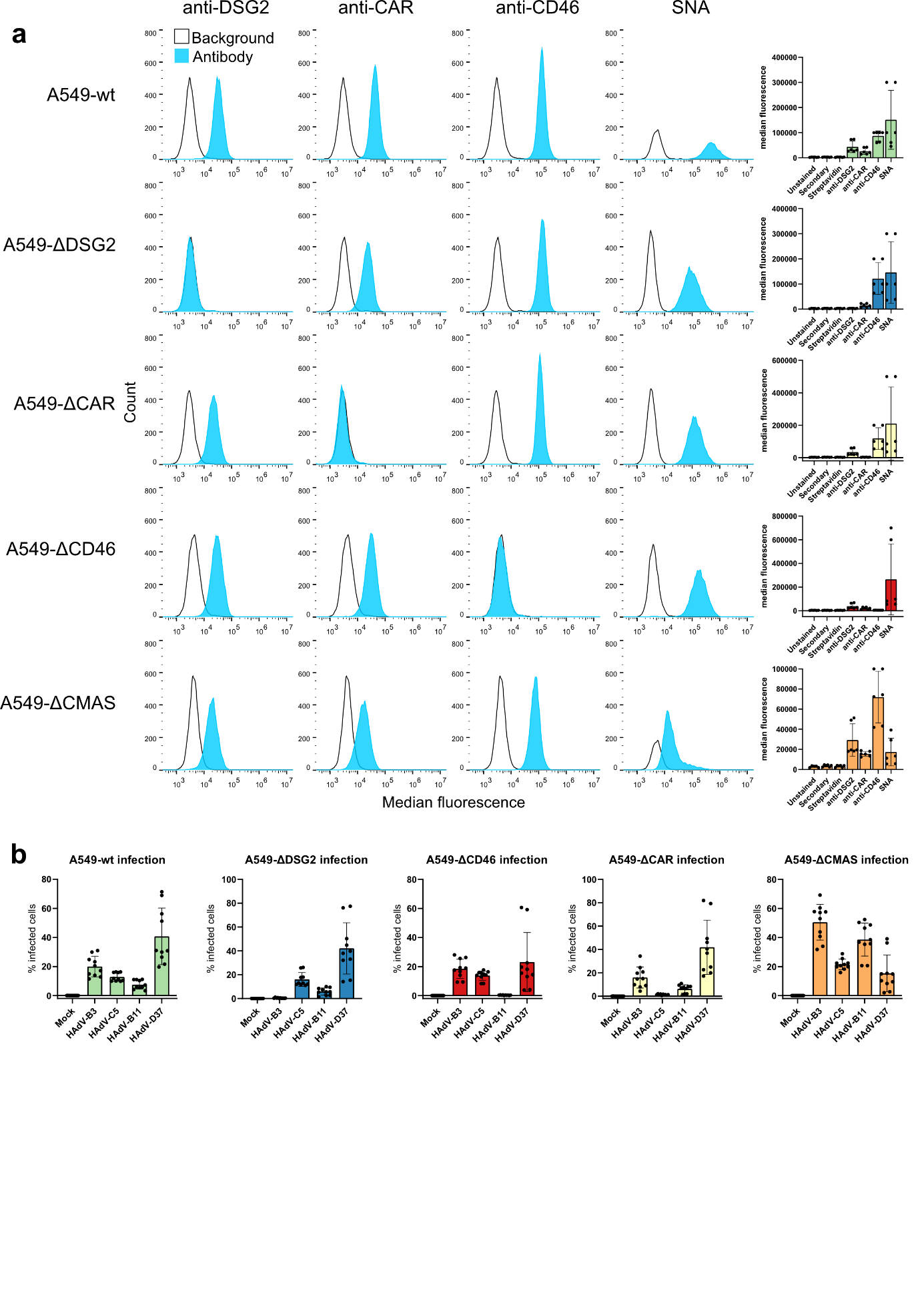


**Supplementary figure 1**. ***a.*** *Cell surface expression of DSG2, CD46, CAR, and α2,6-linked sialic acid in A549-wt, -ΔDSG2, -ΔCD46, -ΔCAR, and -ΔCMAS cells, analysed by flow cytometry. Expression is presented as median fluorescence in histograms (representative from one experiment) and bar graphs (from three independent experiments).* ***b.*** *Infection in A549-wt, -ΔDSG2, -ΔCD46, -ΔCAR, and -ΔCMAS cells with control adenoviruses: HAdV-B3 (DSG2), HAdV-B11 (CD46), HAdV-C5 (CAR), and HAdV-D37 (sialic acid). Data is presented as percent infection (number of infected cells divided by total number of cells). Data in* ***a*** *and* ***b*** *represent the mean ± SD from three independent experiments.*
